# Therapeutic Potential of Hexahydrocurcumin in the Regeneration and Protection of the Retinal Pigment Epithelium

**DOI:** 10.3390/ph18040554

**Published:** 2025-04-09

**Authors:** Ilona Nowak, Robert Kubina, Barbara Strzałka-Mrozik

**Affiliations:** 1 Department of Molecular Biology, Faculty of Pharmaceutical Sciences in Sosnowiec, Medical University of Silesia, 41-200 Sosnowiec, Poland; bstrzalka@sum.edu.pl; 2 Silesia LabMed: Centre for Research and Implementation, Medical University of Silesia, 41-752 Katowice, Poland; rkubina@sum.edu.pl; 3 Department of Pathology, Faculty of Pharmaceutical Sciences in Sosnowiec, Medical University of Silesia, 41-200 Sosnowiec, Poland

**Keywords:** age-related macular degeneration, hexahydrocurcumin, inflammation, oxidative stress, retinal pigment epithelium

## Abstract

Hexahydrocurcumin (HHC), the primary metabolite of curcumin, shows promising therapeutic potential due to its antioxidant and anti-inflammatory properties. The retinal pigment epithelium (RPE) plays a crucial role in maintaining retinal homeostasis; however, its dysfunction—linked to oxidative stress and chronic inflammation—contributes to the progression of degenerative diseases such as age-related macular degeneration (AMD). This review highlights the therapeutic potential of HHC in protecting and regenerating RPE cells. It explores the effects of oxidative stress on the RPE, the mechanisms underlying its damage, and the involvement of reactive oxygen species (ROS) and inflammatory mediators. HHC has demonstrated the ability to modulate these pathways by activating nuclear factor erythroid 2-related factor 2 (NRF2), enhancing antioxidant defenses, and inhibiting pro-inflammatory cytokine production. Preclinical studies suggest that HHC mitigates vascular remodeling and endothelial dysfunction by reducing the expression of transforming growth factor β (TGF-β1) and matrix metalloproteinase-9 (MMP-9). Moreover, HHC improves nitric oxide bioavailability and promotes nitric oxide synthase expression, thereby counteracting oxidative stress-induced vascular damage. Emerging evidence indicates that HHC may be a promising candidate for the treatment of retinal degenerative diseases, particularly those associated with oxidative stress and inflammation. However, further studies, including clinical trials, are essential to confirm its efficacy and elucidate the precise mechanisms underlying HHC’s protective effects on RPE cells.

## 1. Retinal Pigment Epithelium

The retinal pigment epithelium is a single-layer structure composed of polarized, cuboidal, tightly connected, and pigmented cells [1,2]. Tight intercellular junctions are a key feature of the RPE, enabling precise regulation of ion transport [2]. These junctions are predominantly located along the apical side of the RPE layer, where they play a crucial role in maintaining the polarized distribution of proteins between the apical and basolateral membranes. The basolateral membrane of the RPE interfaces with the specialized Bruch’s membrane, further contributing to the regulation of paracellular nutrient transport [1,2].

**Figure 1:**
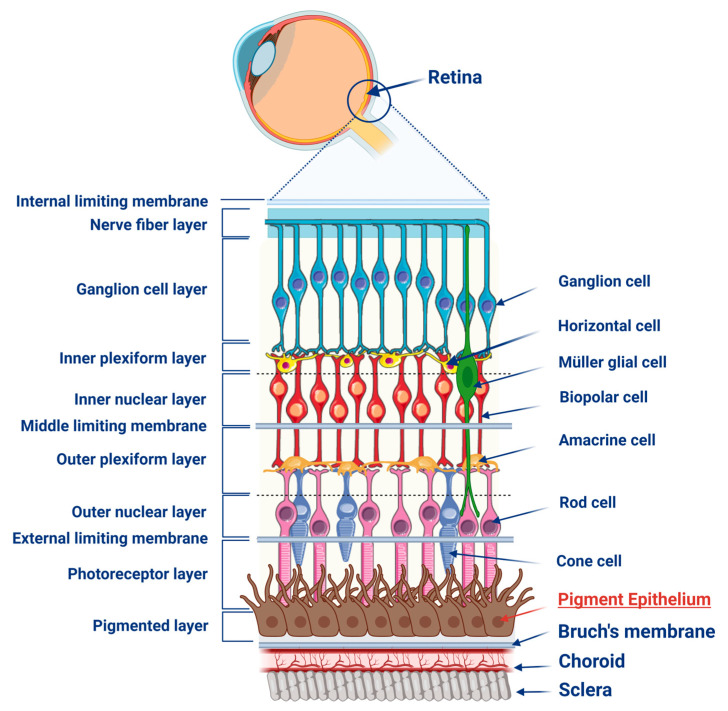
Schematic representation of the structure of the retinal pigment epithelium. The figure was partly generated using Servier Medical Art, provided by Servier and licensed under a Creative Commons Attribution 4.0 unported license.

The RPE is located in the outer part of the retina, positioned between the photoreceptors (rods and cones) and the choroidal vessels, serving as a key component of the blood-retina barrier [3,4]. In the apical regions of RPE cells, numerous cytoplasmic projections extend around the photoreceptors [2]. These projections contain melanin granules, which protect the retina from phototoxicity and cytotoxicity by absorbing free radicals and heavy metal ions [2,5]. The amount of melanin and its functional capacity in the RPE decrease with age, which correlates with increased lipid peroxidation and the accumulation of oxidized cellular components in the RPE [2].The retina of the eye consists of the neural retina, RPE, and choroid [6]. It is composed of multiple layers, with cell bodies organized in three distinct regions [7]. Photoreceptors are located in the outer nuclear layer, while horizontal, bipolar, amacrine, and Müller cells are found in the inner nuclear layer. Amacrine and ganglion cells are situated in the ganglion cell layer [6,8]. The schematic structure of the retina, including its layers, cell types, and the location of the RPE, is presented in Figure 1.

The RPE plays a fundamental role in maintaining normal vision [9]. This structure is essential for ensuring the proper function and viability of photoreceptors in the eye [3,9]. The RPE facilitates the transport of nutrients and oxygen from the choroidal capillaries while regulating metabolite exchange between the retina and blood vessels, thereby promoting retinal homeostasis [4,10]. One of the key processes in photoreceptors is phototransduction—the conversion of light energy into a neural signal [9]. Photoreceptors initiate the phototransduction cascade by triggering the isomerization of 11-cis retinal to all-trans retinal. The RPE plays a crucial role in the visual cycle by recycling all-trans retinal to 11-cis retinal, ensuring the continuous regeneration of visual pigments required for sustained vision [4,10].

In addition, the RPE performs several essential functions, including vitamin A metabolism, regulation of the visual cycle in accordance with circadian rhythms, absorption of scattered and reflected light, phagocytosis of photoreceptor outer segments, and production of the mucopolysaccharide matrix that surrounds the outer retinal segments [10].

## 2. Diseases Associated with the Degradation of the RPE

In the course of disease processes and aging, various changes occur in the retinal pigment epithelium and its surrounding microenvironment. One of the most noticeable alterations is the gradual decline in the number of RPE cells [9]. The degradation of RPE cells plays a crucial role in the pathogenesis of numerous retinal diseases. These processes contribute to the development of conditions such as AMD, pigmentary retinopathy, diabetic retinopathy (DR), and other inflammatory retinal diseases, where oxidative stress and inflammation significantly contribute to RPE and photoreceptor damage [4]. Additionally, the degeneration of photoreceptor cells and the thickening of Bruch’s membrane—associated with the deposition of lipid–protein aggregates and calcification—contribute to a loss of elasticity within the Bruch’s membrane–vascular complex [9]. Changes in RPE pigmentation and morphology have been observed, including the displacement and degradation of melanosomes, as well as the accumulation of lipofuscin granules resulting from impaired lysosomal activity [9]. These alterations can lead to retinal dysfunction and, over time, contribute to the development of pigment-related degenerative diseases, such as pigmentary retinopathy [9]. Furthermore, various pathological conditions can exacerbate pigment degradation in the eye. Uveitis, an intraocular inflammatory condition, may cause pigment dispersion and subsequent damage to retinal structures [11]. Toxoplasma choroiditis, an infectious disease caused by *Toxoplasma gondii*, can lead to focal depigmentation and retinal scarring [12]. Moreover, pigmentary glaucoma is characterized by the dispersion of iris pigment, which may accumulate in the trabecular meshwork and contribute to elevated intraocular pressure [13].

Alterations in RPE pigmentation, such as hypopigmentation (linked to RPE cell atrophy) and hyperpigmentation (resulting from reactive RPE proliferation), serve as biomarkers for various retinal pathologies, including degenerative and inflammatory diseases [10]. A key clinical indicator of impaired proteostasis in the RPE is the accumulation of lipofuscin—a photooxidative pigment—within lysosomes, along with the formation of extracellular drusen deposits between the RPE and Bruch’s membrane. These changes compromise RPE function and increase the risk of degenerative diseases [14].

Jarrett et al. [15] demonstrated that age-related accumulation of lipofuscin is accompanied by increased levels of 8-oxoguanine, a major marker of oxidative DNA damage in the aging retina. This is also associated with mitochondrial DNA (mtDNA) damage, carboxyethylpyrrole (CEP), an oxidative fragment of docosahexaenoic acid, as well as 4-hydroxynonenal (4-HNE) and malondialdehyde (MDA), both of which are byproducts of lipid peroxidation [15]. Morphological changes, such as atrophy, hypertrophy, hyperplasia, migration, or metaplasia, may occur in response to environmental stimuli, mechanical stress, or physical trauma [10].

A deeper understanding of the degenerative mechanisms affecting the RPE is essential for developing new diagnostic strategies and therapeutic approaches aimed at slowing or halting disease progression.

### 2.1. Age-Related Macular Degeneration

Due to constant exposure to light, RPE cells have developed high concentrations of various cytoprotective pigments, including melanin, flavins, and retinoids. These compounds contribute to a yellowish coloration, collectively referred to as macular pigments [14]. The macula, the central region of the retina, is essential for central vision [10]. It plays a crucial role in visual acuity, contrast sensitivity, and color perception. Degeneration of the RPE is a key hallmark of AMD [14].

Degenerative changes in AMD primarily affect the outer retinal layers, including the photoreceptors, the RPE, Bruch’s membrane, and the adjacent choroidal capillary cells responsible for supplying these structures with nutrients [16]. AMD is the leading cause of blindness among older individuals and one of the primary causes of vision loss in people over 50 in developed countries [10,17]. The disease leads to a progressive decline in central vision, significantly impacting patients’ quality of life [10]. Various environmental and genetic factors contribute to the pathogenesis and progression of AMD, including aging, prolonged light exposure, genetic predisposition, and unhealthy lifestyle choices [4].

AMD exists in two primary forms: dry (atrophic; characterized by geographic atrophy and/or drusen; non-exudative) and wet (exudative; associated with the formation of a subretinal neovascular membrane; neovascular) [3,9,10,11,12,13,14]. In atrophic AMD, the early stages are marked by the accumulation of subretinal pigment epithelium deposits, known as drusen, followed in advanced stages by a progressive loss of the RPE, choroid, and photoreceptors [3]. The formation of drusen results in the detachment of underlying structures, creating a physical barrier that disrupts the exchange of nutrients and metabolic waste between the RPE, photoreceptors, and Bruch’s membrane [18]. Currently, there is no approved treatment for non-exudative (dry) AMD—which accounts for approximately 90% of AMD cases—and its precise pathological mechanisms remain incompletely understood [3].

One proposed mechanism underlying AMD development is the age-related phagocytic metabolic failure of post-mitotic (non-renewable) RPE cells [14]. The disease process is thought to be triggered when the redox status of RPE cells shifts from an antioxidant to a pro-oxidant state [16]. Cellular redox homeostasis is regulated by NRF2, a transcription factor that controls genes involved in the cellular response to oxidative stress. NRF2 directly regulates the expression of key oxidative stress pathway genes, such as catalase and superoxide dismutase 1 (SOD1) [17]. In addition, NRF2 modulates the levels of cellular antioxidants, including glutathione (GSH) and thioredoxin. Measuring the expression of NRF2 target genes, along with glutathione and thioredoxin levels, provides a critical readout of cellular redox status and is directly related to AMD pathogenesis [18]. Over the past few decades, numerous studies have confirmed NRF2’s central role in orchestrating the antioxidant response and drug detoxification processes [19]. NRF2 activates pathways that regulate enzymes involved in the synthesis, utilization, and regeneration of reduced GSH. Key NRF2 targets include the catalytic and modulatory subunits of glutamate–cysteine ligase and glutathione synthase, both essential for GSH synthesis [19]. Moreover, several redox cycling enzymes are regulated by NRF2, including thioredoxin, thioredoxin reductase, sulfiredoxin, peroxiredoxin, glutathione peroxidase, and various glutathione S-transferases. Together, these components form a critical defense system that protects cells from oxidative damage.

Oxidation byproducts resulting from excessive ROS production, such as CEP and MDA, have been shown to induce inflammation and AMD-like phenotypes in animal models. Furthermore, levels of 4-HNE—a byproduct of lipid peroxidation—increase with age and are implicated in various pathological processes, with significantly elevated concentrations found in the retina and plasma of patients with AMD [10].

Furthermore, the accumulation of inflammation-associated substances in the surrounding capillaries creates a toxic microenvironment that leads to capillary dysfunction and atrophy. This, in turn, causes hypoxia in RPE cells. In response, these epithelial cells upregulate vascular endothelial growth factor (VEGF), which stimulates the growth of new blood vessels from the choriocapillaris and results in the formation of a neovascular membrane [14].

Proposed mechanisms of RPE cell degeneration in AMD pathogenesis include the activation of the complement cascade, oxidative stress-induced cell death (via necrosis, apoptosis, or pyroptosis) [9,20], mitochondrial dysfunction, and the involvement of αB-crystallins [9]. Identifying the initial molecular changes that trigger these pathological events in the RPE could pave the way for early diagnostic tools and more effective therapeutic strategies for atrophic AMD and other ocular diseases [3].

### 2.2. Diabetic Retinopathy

Diabetic retinopathy is one of the most common retinal vascular diseases, developing as a result of chronic hyperglycemia. It affects approximately 75% of individuals who have had diabetes for at least 15 years [14,16]. The main risk factors include suboptimal glycemic control, arterial hypertension, dyslipidemia, long disease duration, and genetic predisposition [21]. Two main stages of the disease are distinguished: non-proliferative diabetic retinopathy (NPDR) and proliferative diabetic retinopathy (PDR). NPDR is characterized by progressive microvascular changes within the retina, while PDR involves the formation of new blood vessels on the retinal surface or within the optic disk [16,17]. Diabetic macular edema (DME) refers to retinal thickening in the posterior pole and may occur in both NPDR and PDR. The treatment of DR includes preventive strategies, early detection, and ophthalmic interventions such as laser photocoagulation, vitrectomy, and intravitreal pharmacotherapy—all aimed at minimizing the risk of vision loss [22].

Hyperglycemia leads to excessive production of ROS in the mitochondria of the retinal pigment epithelium, resulting in lipid peroxidation, DNA damage, and the activation of pro-inflammatory pathways, such as NF-κB. Increased ROS production contributes to microglial activation and macrophage infiltration, further exacerbating chronic inflammation [14]. Chronic microinflammation in DR is sustained by cytokines such as interleukin 6 and 8 (IL-6, IL-8), and monocyte chemoattractant protein-1 (MCP-1), C-C Motif Chemokine Ligand 2 (CCL2), which affect various retinal cell types. IL-6 acts on astrocytes, leading to dysfunction of the inner blood–retina barrier (BRB), while IL-8 and MCP-1 promote the recruitment of neutrophils and monocytes to the retina. Additionally, leukocytes and microglial cells play a crucial role in the progression of DME [4].

Dysfunction of the RPE in diabetes is a crucial factor in the pathogenesis of DR, extending beyond the traditional perception of the disease as solely a vascular disorder [23]. Autophagy in the RPE is an essential homeostatic process that facilitates the removal of damaged organelles and abnormal proteins. Under hyperglycemic conditions, this process becomes disrupted, leading to the accumulation of damaged mitochondria and increased oxidative stress. Studies have shown that the expression of autophagy markers such as microtubule-associated protein 1A/1B-light chain 3 (LC3-II) and Beclin-1 is reduced, indicating dysregulation of this process in diabetes [17].

## 3. Pathology of RPE Degradation

The RPE plays a key role in maintaining retinal homeostasis, but its function can be impaired by various factors, most notably oxidative stress. This structure is particularly vulnerable to oxidative damage due to its high metabolic activity [4,10,18]. The elevated metabolic activity of the RPE is attributed, among other factors, to the interaction of a single epithelial cell with multiple photoreceptors, the continuous phagocytosis of photoreceptor outer segments (POS), the abundance of mitochondria, and the need to provide metabolic support to the retina [4,9,20].

Additionally, another crucial function of the RPE is the phagocytosis of exfoliated POS from photoreceptors, which contain photosensitive groups, various oxidants, and unsaturated fatty acids. This process further contributes to increased ROS production in the RPE [4]. ROS include the superoxide radical (O_2_•−), hydrogen peroxide (H_2_O_2_), the hydroxyl radical (OH_2_), and singlet oxygen (^1^O_2_) [4]. Under physiological conditions, ROS and reactive nitrogen species (RNS) can act as second messengers in signal transduction and play a key role in maintaining normal cellular function [4,9]. However, excessive ROS and RNS production can disturb the intracellular redox balance, leading to the peroxidation of cellular components such as lipids, proteins, and nucleic acids, ultimately resulting in mitochondrial dysfunction [4]. The RPE functions in an environment with elevated levels of ROS, which are produced as a result of intense metabolic processes, UV exposure, and constant photochemical reactions occurring in the retina during vision [4,18,20]. Sources of cellular ROS can originate from mitochondria or be influenced by environmental factors such as cigarette smoke [24].

To reduce oxidative stress and support the protection of RPE cells, lifestyle-based strategies are strongly recommended. A key component of these strategies is ensuring adequate intake of antioxidant nutrients—both through dietary sources and by promoting endogenous synthesis of protective compounds such as glutathione, coenzyme Q10, and ubiquinol [25]. Diets rich in antioxidants—such as lutein, zeaxanthin, vitamin C, vitamin E, and zinc—can help preserve retinal function and mitigate the damaging effects of reactive oxygen species. Omega-3 fatty acids, found in oily marine fish such as salmon, tuna, and mackerel, also exhibit anti-inflammatory properties that may benefit RPE cell health [26]. Regular consumption of leafy green vegetables, including spinach, broccoli, and kale, provides important nutrients like lutein that support retinal structure and function [27].

Polyphenols have been shown to reduce RPE cell necrosis, lens opacity, and breakdown of the blood–retinal barrier [27]. Resveratrol, a polyphenolic antioxidant and phytoalexin found in various fruits and plant-based foods, has demonstrated protective effects by inhibiting excessive VEGF production in RPE cells under oxidative stress and preventing blue-light-induced cell death [28]. Additionally, reducing modifiable risk factors—such as smoking—is critical for protecting vision. Cigarette smoke contributes to excessive free radical production, and hydroquinone, one of its components, has been implicated in RPE cell degeneration [27].

Lykkesfeldt et al. [29] reported that cigarette smoke contains various potent oxidants that can deplete ascorbic acid and sulfhydryl groups in RPE proteins, leading to an imbalance in the antioxidant system and oxidative damage to DNA, lipids, and proteins. Additionally, a high-fat diet has also been identified as a risk factor for inducing oxidative stress in the RPE [18]. Datta et al. [24] demonstrated that long-term feeding of mice with a high-fat diet resulted in the suppression of Wingless (Wnt) signaling in the RPE, which impaired an antioxidant network primarily regulated by NRF2. Mitochondria and the endoplasmic reticulum (ER) have been implicated in oxidative stress-related diseases [10]. Cano et al. [30] demonstrated that oxidative stress induced by cigarette smoke extract leads to mitochondrial dysfunction in the RPE, despite the activation of the protective unfolded protein response (UPR). Although the UPR is activated in response to oxidative stress, mitochondria remain vulnerable to damage, which may contribute to disease development [30].

Mitochondrial ROS generators include cytochrome P450, nicotinamide adenine dinucleotide phosphate oxidases (NADPH oxidases, NOX), and xanthine oxidase (XO). However, the primary source of ROS is the mitochondrial electron transport chain, which plays a crucial role in intracellular signaling. The exact mechanism of ROS accumulation in cells remains unclear, although it has been suggested that mutations in the *SOD2* gene, exposure to visible light, and the accumulation of lipofuscin may contribute to this process [9].

As the retina continuously performs photochemical reactions to enable vision, it requires a substantial amount of oxygen and undergoes intensive oxidation processes to generate energy. When exposed to blue light, numerous peroxides and free radicals are easily formed, further damaging retinal cells [31]. Several studies by Grimm et al. [32,33] demonstrated that blue light can induce ROS formation and trigger photoreceptor death, with this effect being dependent on rhodopsin [2]. Similarly, Kuse et al. [34] showed that high-energy blue light can induce photoreceptor apoptosis, promote ROS production, and cause protein alterations.

In addition, the retina contains a significant amount of unsaturated fatty acids, which are closely linked to free radical production. As a result, lipid peroxidation in the retina occurs readily, leading to oxidative DNA damage [31]. Oxidative processes not only cause damage in the macular area but also affect the lens, vitreous body, and capillaries within the eye [31]. In summary, oxidative stress is associated with nearly all eye diseases, including macular degeneration, vitreous body opacities, cataracts, and dry eye syndrome [35,36].

**Figure 2:**
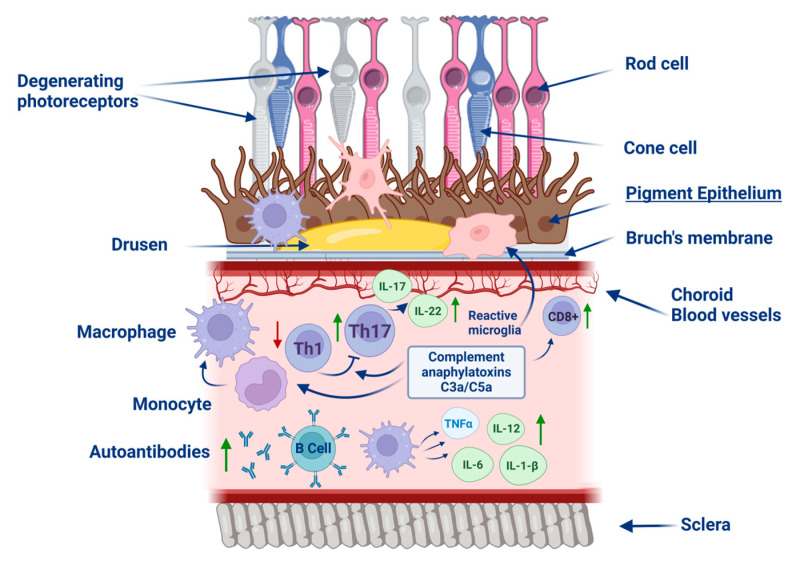
Schematic representation of the role of macrophage-regulated immune inflammation in the pathogenesis of age-related macular degeneration. The factors involved in the immune response include macrophages, monocytes, autoantibodies, lymphocytes (Th1, Th17, B, CD8+), and activated microglia. Inflammatory mediators such as interleukins are also highlighted. The role of complement anaphylatoxins, C3a and C5a, which may contribute to inflammatory and degenerative processes, is also emphasized. The figure was partly generated using Servier Medical Art, provided by Servier and licensed under a Creative Commons Attribution 4.0 unported license.

The presence of immune-related proteins, particularly complement system components, within drusen suggests that inflammation in the subretinal space plays a crucial role in the pathological changes associated with AMD, ultimately leading to the degeneration of RPE cells (Figure 2) [37].

The complement system is a key element of the innate immune response, responsible for regulating excessive immune activation and protecting tissues from unnecessary inflammation. Because the eye is an immune-privileged organ, complement activity within it is tightly controlled, with only limited quantities of its components present [38]. The retinal pigment epithelium and retinal microglia are the primary sources of these proteins in the eye. Disruption of local immune homeostasis may contribute to the development of various ocular diseases, including age-related conditions such as AMD [39].

Activation of the complement system occurs in response to injury, triggering the release of cellular signals—such as cytokines, chemokines, and growth factors—that influence neighboring cells. This activation initiates a cascade of pathophysiological responses, including the clearance of pathogens and damaged cells, the formation of membrane attack complexes on pathogens, and the promotion of inflammation [9].

It has been demonstrated that the RPE can produce and release various cytokines and chemokines, including RANTES, monocyte chemotactic protein-1, IL-6, and IL-8, thereby triggering an inflammatory response [14].

The complement system can be activated through the classical pathway (CP), the mannose-binding lectin pathway, or the alternative pathway. All three mechanisms converge on the activation of complement component 3 (C3) convertase, which cleaves the C3 complex and subsequently leads to the activation of complement component 5 (C5) convertase. This process results in the formation of the C5bC9 complex, also known as the membrane attack complex (MAC), which plays a crucial role in cell lysis and the promotion of inflammation. Additionally, complement cascade fragments—such as C3a released during C3 cleavage and C5a generated during C5 cleavage—function as essential anaphylatoxins that facilitate anaphylactic responses, chemotaxis, and immune regulation. C3b contributes to opsonization, enhancing the binding of molecules, microbes, or apoptotic cells to immune cell receptors, thereby promoting the phagocytosis of antigens and apoptotic cells. Under physiological conditions, the MAC is effectively degraded by protein S (vitronectin), maintaining homeostasis and preventing uncontrolled cytolysis [9].

## 4. Curcuminoids—Biological Properties and Therapeutic Potential

Compounds of natural origin with potential antioxidant and anti-inflammatory properties are of significant interest and promise. Turmeric is one of the most popular medicinal herbs, particularly in traditional Chinese and Indian subcontinental medicine [40]. Recently, bioactive curcuminoids, including curcumin—a lipophilic polyphenol—have garnered increasing attention due to their anticancer, antimicrobial, anti-inflammatory, and anti-aging effects [41].

### 4.1. Curcumin

One such compound is the yellow pigment curcumin, chemically known as [1,7-bis(4-hydroxy-3-methoxyphenyl)-1,6-heptadiene-3,5-dione] [31,40,41]. Curcumin is the main constituent of turmeric, which is obtained—among other methods—by extracting the rhizomes of *Curcuma longa* L., a member of the ginger family [40,42]. The structural formula of curcumin features two phenolic groups and exists in two tautomeric forms: keto and enolic. It is practically insoluble in neutral and acidic aqueous solutions at room temperature, but it is more soluble in organic solvents such as methanol, ethanol, acetone, or dimethyl sulfoxide, as well as in oils [31,42]. Its solubility in water can be enhanced by adding surfactants such as sodium dodecyl sulfate, polysaccharides, polyethylene glycol, or cyclodextrins [42].

Curcumin exhibits a broad spectrum of pharmacological activities, including anticancer, antioxidant, anti-inflammatory, and antimicrobial properties. It is effective against *Staphylococcus aureus*, *Salmonella paratyphi*, and *Mycobacterium tuberculosis*, among others [42,43]. It also shows therapeutic potential in treating various conditions, such as cardiovascular diseases, diabetes, arthritis, and both neurological and neurodegenerative disorders [34]. Additionally, curcumin interacts with a variety of molecules that regulate numerous biological processes, including protein kinases, xanthine oxidase, human serum albumin, and proteases of human immunodeficiency virus (HIV-1 and HIV-2) [40]. In recent years, due to its effects on oxidative stress, angiogenesis, and inflammatory processes, it has also begun to be used in the treatment of ocular diseases [43].

Curcumin has potent antioxidant activity, protecting cells from protein carbonylation, lipid peroxidation, and altered mitochondrial permeability [44]. Its antioxidant activity has been shown to be about 10 times higher than that of vitamin E. Curcumin is one of the most potent antioxidants with a high potential to reduce age-related cellular damage induced by ROS generation. Due to the presence of phenolic groups in its chemical structure, it exhibits strong hydrogen-transferring antioxidant activity [44]. Several studies have shown that curcumin prevents lipid peroxidation and enhances antioxidant activity, including glutathione-S-transferase (GST), GSH, superoxide dismutase, and glutathione peroxidase (GPx) in various types of cancer in different organs [44]. Curcumin exhibits anti-inflammatory effects by inhibiting the activity of cyclooxygenase-2 (COX-2), lipoxygenase (LOX), the inflammasome, and inducible nitric oxide synthase (iNOS). Moreover, it reduces the levels of pro-inflammatory cytokines such as interleukins (IL-2, IL-6, IL-8, IL-12) and tumor necrosis factor-alpha (TNF-α), as well as chemokines, including macrophage inhibitory protein (MIP) and MCP-1. Additionally, curcumin decreases the activity of mitogen-activated protein kinases (MAPK) and Janus kinases (JAK) [45].

Curcumin has demonstrated potential in modulating key biochemical pathways involved in DR. It may inhibit the activation of protein kinase C, thereby reducing inflammation, oxidative stress in the retina, and associated vascular abnormalities [46,47]. Both curcumin and its metabolites are also believed to influence the polyol pathway by decreasing oxidative stress and limiting the accumulation of sorbitol, a hallmark of this metabolic route. Furthermore, curcumin can modulate the hexosamine pathway by inhibiting the formation of advanced glycation end products (AGEs), which are closely associated with retinal damage in diabetes [46,47]. In addition to its antioxidant effects, curcumin has been reported to reduce nitrotyrosine levels in the retina, potentially preventing structural damage caused by the accumulation of this molecule [48]. Despite these promising mechanisms, the therapeutic application of curcumin in the treatment of DR remains in the early stages of research and warrants further investigation.

Analysis of a number of clinical and preclinical studies has shown that curcumin can be used as a therapeutic agent for the treatment of various ocular diseases [31]. Table 1 shows selected clinical studies using curcumin.

Unfortunately, curcumin is an unstable compound and can degrade under physiological conditions via hydrolysis or enzymatic reactions [42]. It is rapidly metabolized both in cell culture and in vivo, primarily through reduction and subsequent conjugation [43].

**Figure 3:**
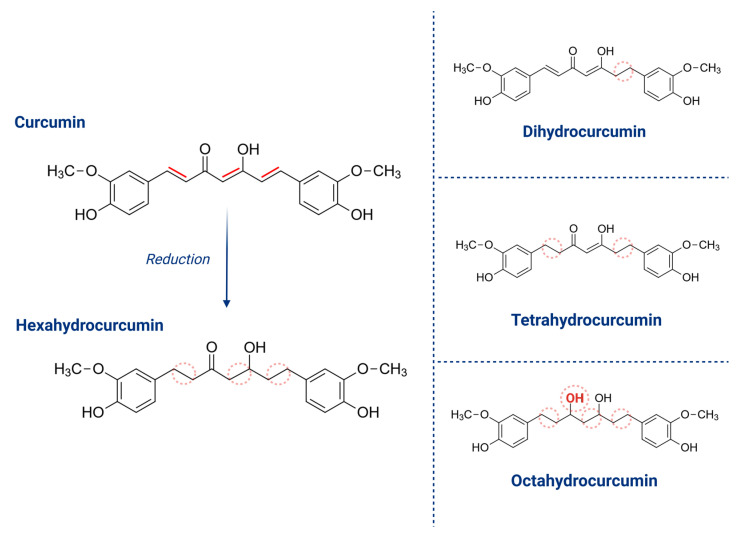
Structure of curcumin and its metabolites.

When administered orally, curcumin becomes conjugated with glucuronic acid and sulfuric acid, primarily forming the glucuronide of hexahydrocurcumin. After intravenous or intraperitoneal administration, it undergoes reduction, leading to the formation of tetrahydrocurcumin and hexahydrocurcumin, which are the two main metabolites of curcumin in body fluids [40,43]. It is also converted into octahydrocurcumin in the liver, as well as in other organs and tissues, with dihydrocurcumin appearing as a minor metabolite [31,33]. Figure 3 illustrates the structure of curcumin and its metabolites. Additionally, curcumin is metabolized in the intestinal mucosa via phase I metabolism [42].

Curcumin exhibits several physicochemical and pharmacokinetic limitations [40]. It is chemically unstable at neutral and slightly alkaline pH values [31,40,42,43]. Moreover, curcumin is rapidly metabolized in the bloodstream and has low bioavailability due to its poor absorption and limited passage through the gastrointestinal tract, which restricts its practical clinical application [31,40,43]. Even after administering high oral doses, only low concentrations of curcumin are detected in plasma, urine, and peripheral tissues [40].

### 4.2. Hexahydrocurcumin

**Figure 4:**
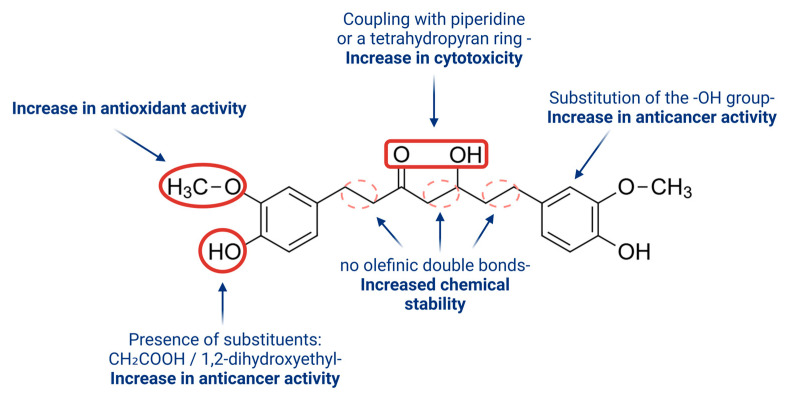
Structure dependence of hexahydrocurcumin on its biological activity.

The main metabolites of curcumin are tetrahydrocurcumin and hexahydrocurcumin [44]. Hexahydrocurcumin ((5S)-hydroxy-1,7-bis(4-hydroxy-3-methoxyphenyl)heptan-3-one) exhibits bioactivity comparable to or even greater than that of curcumin in both in vitro and in vivo studies [23,41]. Moreover, HHC is more stable than curcumin at pH 7.4 and can be produced in significant quantities by the catalytic hydrogenation of curcumin [40,43]. Due to its shared phenolic and diketone groups, HHC may demonstrate similar biological and pharmacological properties as curcumin. Notably, the absence of olefinic double bonds in HHC may enhance its stability in the human body after metabolism, potentially extending its antioxidant effect. Additionally, the increased number of hydroxyl groups and the presence of a phenyl fragment might contribute to its stronger antioxidant properties [44]. Figure 4 illustrates the relationship between the structure of hexahydrocurcumin and its biological activity.

In vitro and in vivo studies have demonstrated that HHC possesses protective physiological and pharmacological properties similar to those of curcumin, including radical scavenging, anticancer, anti-inflammatory, and cardiovascular protective effects [31,44,49].

Curcumin has been shown to modulate the NRF2 pathway by inducing the expression of cytoprotective proteins in an NRF2-dependent manner. Under conditions of oxidative stress, curcumin activates NRF2, leading to the upregulation of antioxidant enzymes such as gamma-glutamylcysteine ligase, a key enzyme in glutathione synthesis. It has been reported that curcumin protects renal tubular epithelial cells from hydrogen peroxide-induced oxidative damage by enhancing NRF2-mediated antioxidant defenses [50].

Similar mechanisms have been proposed for HHC, which may also induce cytoprotective proteins through NRF2 activation. HHC is suggested to upregulate antioxidant enzymes in a manner analogous to curcumin; however, current evidence is limited, and further investigation is required to fully understand this pathway [50].

The expression of NRF2-regulated cytoprotective genes is controlled by three key elements: the antioxidant response element (ARE), NRF2, and Kelch-like ECH-associated protein 1 (Keap1). Keap1, which acts as a cytoplasmic repressor of NRF2, senses oxidative stress through modifications to its cysteine residues. These changes cause a conformational shift in Keap1, leading to the release of NRF2 [50]. Once released, NRF2 translocates into the nucleus, where it dimerizes with Maf transcription factors and binds to ARE sequences, initiating the transcription of genes involved in antioxidant defense. Additionally, Keap1 plays a critical role in the ubiquitination and proteasomal degradation of NRF2, regulating its intracellular levels under basal conditions. Beyond its role in oxidative stress response, NRF2 also regulates genes involved in lipid metabolism, underscoring its broader function in cellular homeostasis [51].

Moreover, HHC exhibits enhanced antioxidant activity, particularly in its ability to inhibit lipid peroxidation and prevent red blood cell hemolysis. Potential molecular targets of HHC include inducible COX-2, VEGF, dexamethasone, amyloid precursor protein, and β-secretase cleaving enzymes [43].

Regarding the effect of HHC on retinal pigment epithelial cells in the context of regeneration, it is important to clarify that regeneration in this case does not imply the restoration of structural tissue integrity. Rather, it refers to mechanisms that support the maintenance of cellular function and the stability of existing cells. The antioxidant and anti-inflammatory properties of HHC play a key role in this process, as they may help protect RPE cells from oxidative stress and chronic inflammation—factors that contribute significantly to cellular dysfunction and degeneration.

Although the therapeutic potential and biological properties of hexahydrocurcumin are under active investigation, current data on its bioanalysis and pharmacokinetics remain limited. A structural and functional comparison between curcumin and its reduced derivative, HHC, is presented in Table 2.

Chaiyasaeng et al. [51] conducted a comparative analysis of the pharmacokinetics and tissue distribution of HHC following intraperitoneal and oral administration in mice. The half-life of HHC observed in their study was longer than that of curcumin, which has a reported half-life of 32.7 min after oral administration in rats.

Tissue distribution analysis revealed that, following intraperitoneal administration, HHC was primarily concentrated in the liver and kidneys, with minimal accumulation in the brain. This suggests that the hydrophobic nature of HHC may limit its ability to cross the blood–brain barrier [51]. Despite these promising findings, the limitations and potential side effects of HHC remain insufficiently characterized. The lack of comprehensive safety and long-term toxicity studies continues to be a major barrier to its potential clinical application [49].

## 5. In Vitro and In Vivo Studies of Hexahydrocurcumin

The anti-angiogenic effect of HHC as a COX-2 inhibitor has been demonstrated in corneal neovascularization (CorNV). In a study conducted by Kuo et al. [52], 24 rats with genetically induced CorNV received sub-conjunctival administration of 1 μg HHC, which effectively inhibited angiogenesis by reducing the expression of key pro-angiogenic factors, including basic fibroblast growth factor (bFGF) and VEGF. This effect became evident as early as six days after administration.

Additionally, a significant decrease in HLA-DR expression was observed, suggesting an immunomodulatory effect of HHC. These findings indicate that HHC may serve a therapeutic role in CorNV treatment by acting as a VEGF pathway inhibitor and exhibiting potential anti-inflammatory and immunosuppressive properties [51].

Lin et al. [31] reported that UVB radiation (wavelength 280–315 nm) damages RPE cells by inhibiting cell proliferation, disrupting mitochondrial function, causing cellular DNA damage, and inducing apoptosis. Their study utilized both the human RPE cell line ARPE-19 and primary mouse RPE cells, which were exposed to blue light to simulate phototoxic stress. The effects of HHC on RPE cell proliferation and the activation of intracellular protective mechanisms were examined. In addition, next-generation RNA sequencing (NGS) was employed to identify molecular pathways associated with HHC’s protective role following blue light exposure. HHC was found to be more effective than curcumin in mitigating blue-light-induced damage. It reduced the production of reactive oxygen species (ROS), thereby limiting oxidative damage, alleviated the accumulation of misfolded proteins, and protected RPE cells from apoptosis. Despite these promising results, the underlying mechanisms of HHC’s protective actions remain unclear, and its effects on RPE cells are still largely unexplored [31]. This research gap presents a promising direction for future studies, particularly in the context of retinal protection and therapeutic development.

HHC was also evaluated for its effects on high blood pressure, vascular dysfunction, and remodeling induced by N-nitro-L-arginine methyl ester (L-NAME) in rats [53]. Male Wistar rats received L-NAME (40 mg/kg) in drinking water for seven weeks. During the final three weeks, they were orally administered HHC (20, 40, or 80 mg/kg) or enalapril (10 mg/kg). Blood pressure was measured weekly [53].

L-NAME induction resulted in the development of hypertension, vascular dysfunction, and remodeling, characterized by vessel wall thickening, increased cross-sectional area, and collagen deposition in the aorta. Hypertensive rats also exhibited excessive expression of Nuclear Factor kappa-light-chain-enhancer of activated B cells (NF-κB), Vascular Cell Adhesion Molecule-1 (VCAM-1), and Intercellular Adhesion Molecule-1 (ICAM-1), along with elevated levels of TNF-α. Increased phosphorylation of protein kinases involved in oxidative stress and inflammatory signaling pathways (p-ERK1/2, p-JNK, p-p38) was also observed, along with elevated expression of TGF-β1, MMP-9, and type I collagen. Additionally, oxidative stress markers were elevated, while plasma nitric oxide levels were reduced. The expression of NOS genes in the aorta was downregulated, whereas synthetic protein production was increased. Treatment with HHC or enalapril mitigated these changes [53].

The results indicate that HHC exhibits antihypertensive effects by improving vascular function and inhibiting vascular remodeling, likely due to its antioxidant and anti-inflammatory properties. HHC reduced L-NAME-induced oxidative stress, enhanced antioxidant activity, and suppressed the expression of TGF-β1, MMP-9, and type I collagen (COL1). Its anti-inflammatory action involved the suppression of the NF-κB pathway, leading to decreased TNF-α production and reduced ICAM-1 and VCAM-1 expression [53].

Jearjaroen et al. [54] investigated the effects of HHC on demyelination and cognitive impairment in rats with chronic cerebral hypoperfusion (CCH). The CCH model was induced by bilateral common carotid artery occlusion (BCCAO) for 29 days. This condition resulted in significant myelin damage, activation of A1-type astrocytes, and reactive microglia, which increased the production of pro-inflammatory cytokines and led to cognitive impairment. HHC administration improved myelin integrity, inhibited the activation of harmful astrocytes and microglia, and enhanced the expression of regenerative factors such as IGF-1 and TGM2, contributing to improved cognitive function in CCH rats [54].

These findings suggest that HHC may be a promising therapeutic agent for treating white matter damage and cognitive deficits associated with chronic cerebral hypoperfusion [54].

The therapeutic potential of HHC and related compounds is further supported by the work of Sudarshan et al. [55], who reported significant advances in the synthesis of diarylheptanoids, including curcumin derivatives. Diarylheptanoids are a class of plant secondary metabolites predominantly found in species of the genera *Curcuma*, *Alpinia*, *Zingiber*, and *Alnus*, characterized by two aromatic rings connected by a seven-carbon chain. These compounds have demonstrated a broad spectrum of biological activities, including anti-inflammatory, antioxidant, anticancer, antiviral, hepatoprotective, and neuroprotective properties. Their antiproliferative potential has been highlighted in studies showing inhibition of human cancer cell lines such as MCF-7 (breast cancer) and HepG2 (hepatocellular carcinoma) [55].

Furthermore, Sudarshan et al. [56] reported the successful synthesis of asymmetric linear diarylheptanoids containing a syn-1,3-diol unit and their enantiomers. The cytotoxic potential of these compounds was evaluated using the MTT assay in HeLa cells. The synthetic strategy employed involved a combination of Wittig acylation and olefination, underlining the potential of these compounds as promising candidates for further investigation in therapeutic development [56].

## 6. Future Perspectives

Hexahydrocurcumin exhibits numerous pharmacological properties, and its impact on oxidative stress and inflammation is well documented. This may hold significant importance in the treatment of diseases associated with RPE degeneration.

Previous studies have demonstrated that HHC exerts a protective effect on RPE, particularly in the context of damage induced by blue light. Its mechanism of action involves promoting autophagy, reducing oxidative stress, and mitigating endoplasmic reticulum stress. These properties suggest HHC’s potential antioxidant activity in RPE, although the precise molecular pathways remain incompletely understood. Recent findings indicate that HHC may also influence mitochondrial function, enhancing cellular energy metabolism and promoting cell survival under oxidative stress conditions. This aspect is particularly relevant given that mitochondrial dysfunction is a key factor in the pathogenesis of many retinal degenerative diseases, including AMD. By improving mitochondrial homeostasis, HHC could potentially delay disease progression and support cellular repair mechanisms. Comprehensive studies on the molecular mechanisms underlying HHC’s effects in RPE cells are still limited. Future research should focus on identifying key signaling pathways, such as those involved in mitochondrial biogenesis, antioxidant defense systems, and inflammatory responses. Focusing research on specific cell lines may provide key insights into their protective properties and potential therapeutic applications. Moreover, identifying the early molecular changes that trigger pathological events in RPE could contribute to the development of more effective diagnostic and therapeutic strategies, particularly for the treatment of atrophic AMD and other degenerative retinal diseases. Given its favorable safety profile and natural origin, HHC also presents an attractive candidate for combination therapies that aim to enhance the efficacy of existing treatments.

Therefore, further research on HHC—including studies involving both animal models and clinical trials—is necessary to fully evaluate its potential as a therapeutic candidate for supporting the treatment of degenerative retinal diseases. Additionally, exploring optimal dosing strategies, delivery methods (such as nanoformulations), and potential synergistic effects with other compounds may further enhance its clinical utility.

## Figures and Tables

**Table 1:** Selected clinical trials of using curcumin as a therapeutic agent for eye disorders found at https://clinicaltrials.gov (accessed on 10 February 2025).

Research Title	Intervention/Treatment	Time Frame	Enrollment	Phase	Status	NCT Number
Effect of Oral Curcumin Supplementation in Dry Age-related Macular Degeneration (AMD) Patients	Drug: Longvida Curcumin Other: Placebo	baseline, 3-month, 6-month, 12-month timepoints	10 (Actual)	Early Phase 1	Completed	NCT04590196
A Study to Evaluate the Safety and Efficacy of RQC for AMD	Drug: 100 mg Resveratrol, 120 mg Quercetin, 1000 mg Curcumin (RQC) Drug: 1000 mg Curcumin	24 months	150 (Estimated)	Phase 2	Unknown status	NCT05062486
Anti-inflammatory Effect of Curcumin, Homotaurine, Vitamin D3 on Human Vitreous in Patients With Diabetic Retinopathy	Drug: 0.5 µM i 1 µM Curcumin, 100 µM Homotaurine, 50 nM Vitamin D3 Other: control	7 days	25 (Actual)	-	Completed	NCT04378972

**Table 2:** A structural and functional comparison between curcumin and its reduced derivative, HHC.

Aspect	Hexahydrocurcumin (HHC)	Curcumin	Take-Home Messages
Chemical structure	Reduced form of curcumin (saturated bonds, lacks double bonds)	Natural polyphenol with conjugated double bonds and a diketone moiety	Structural modification improves stability and bioavailability
Chemical stability	Higher—resistant to oxidation, light, and alkaline conditions	Unstable—degrades in light, heat, and alkaline environments	HHC is significantly more stable than curcumin
Bioavailability	Higher—better absorption and systemic presence	Very low (<1%)	HHC is more efficiently absorbed, enhancing its therapeutic potential
Antioxidant properties	Strong—often stronger than curcumin	Strong	HHC maintains or surpasses curcumin’s antioxidant capacity
Anti-inflammatory activity	Effective—comparable or superior to curcumin	Proven anti-inflammatory effects	HHC may provide more potent or sustained anti-inflammatory effects
Anticancer potential	Promising—in vitro studies show activity	Well-documented anticancer properties	Further studies needed, but HHC shows strong preliminary potential
Neuroprotective effects	High—protects neurons and combats oxidative stress	Present, but limited by low bioavailability	HHC may offer better support in neurodegenerative conditions
Therapeutic application	Potentially broader—due to improved pharmacokinetics	Known uses limited by poor stability and absorption	HHC is a strong candidate for next-generation curcumin-based therapeutics

## Data Availability

No new data were created or analyzed in this study. Data sharing is not applicable.

## Abbreviations

4-HNE	4-hydroxynonenal
AMD	age-related macular degeneration
BCCAO	bilateral common carotid artery occlusion
bFGF	basic fibroblast growth factor
BRB	blood-retina barrier
C3	complement component 3
CCH	chronic cerebral hypoperfusion
CCL2	C-C motif chemokine ligand 2
CEP	carboxyethylpyrrole
COL1	type I collagen
CorNV	corneal neovascularization
COX-2	cyclooxygenase-2
CP	classical pathway
DME	diabetic macular edema
DR	diabetic retinopathy
ER	endoplasmic reticulum
GPx	glutathione peroxidase
GSH	glutathione
GST	glutathione-S-transferase
HHC	hexahydrocurcumin
HIV	human immunodeficiency virus
ICAM-1	intercellular adhesion molecule-1
IL	interleukin
iNOS	inducible nitric oxide synthase
JAK	janus kinases
LC3-II	microtubule-associated protein 1A/1B-light chain 3 II
LOX	lipoxygenase
MAC	membrane attack complex
MAPK	mitogen-activated protein kinases
MCP-1	monocyte chemoattractant protein-1
MDA	malondialdehyde
MIP	macrophage inhibitory protein
MMP-9	matrix metalloproteinase-9
mtDNA	mitochondrial DNA
NADPH	nicotinamide adenine dinucleotide phosphate
NF-κB	nuclear factor kappa-light-chain-enhancer of activated B cells
NOX	nicotinamide adenine dinucleotide phosphate oxidases
NPDR	non-proliferative diabetic retinopathy
NRF2	nuclear factor erythroid 2-related factor 2
PDR	proliferative diabetic retinopathy
POS	photoreceptor outer segments
RNS	reactive nitrogen species
ROS	reactive oxygen species
RPE	retinal pigment epithelium
SOD1	superoxide dismutase 1
TGF-β1	transforming growth factor β
TNF-α	tumor necrosis factor-alpha
UPR	unfolded protein response
VCAM-1	Vascular Cell Adhesion Molecule-1
VEGF	vascular endothelial growth factor
Wnt	suppression of Wingless
XO	xanthine oxidase

## References

[1] Strauss O., Kolb H., Fernandez E., Jones B., Nelson R. (1995). The Retinal Pigment Epithelium. Webvision: The Organization of the Retina and Visual System.

[2] Kaufmann M., Han Z. (2024). RPE Melanin and Its Influence on the Progression of AMD. Ageing Res. Rev..

[3] Chen X., Wang Y., Wang J.-N., Cao Q.-C., Sun R.-X., Zhu H.-J., Zhang Y.-R., Ji J.-D., Liu Q.-H. (2022). m6A Modification of circSPECC1 Suppresses RPE Oxidative Damage and Maintains Retinal Homeostasis. Cell Rep..

[4] Zhang S.-M., Fan B., Li Y.-L., Zuo Z.-Y., Li G.-Y. (2023). Oxidative Stress-Involved Mitophagy of Retinal Pigment Epithelium and Retinal Degenerative Diseases. Cell. Mol. Neurobiol..

[5] Kwon Y.-S., Zheng M., Zhang A.Y., Han Z. (2022). Melanin-like Nanoparticles as an Alternative to Natural Melanin in Retinal Pigment Epithelium Cells and Their Therapeutic Effects against Age-Related Macular Degeneration. ACS Nano.

[6] Cell Types of the Human Retina and Its Organoids at Single-Cell Resolution Cowan et al—EGA European Genome-Phenome Archive. https://ega-archive.org/studies/EGAS00001004561.

[7] Masland R.H. (2001). The Fundamental Plan of the Retina. Nat. Neurosci..

[8] Masland R.H. (2012). The Neuronal Organization of the Retina. Neuron.

[9] Somasundaran S., Constable I.J., Mellough C.B., Carvalho L.S. (2020). Retinal Pigment Epithelium and Age-Related Macular Degeneration: A Review of Major Disease Mechanisms. Clin. Exp. Ophthalmol..

[10] Tong Y., Wu Y., Ma J., Ikeda M., Ide T., Griffin C.T., Ding X.-Q., Wang S. (2023). Comparative Mechanistic Study of RPE Cell Death Induced by Different Oxidative Stresses. Redox Biol..

[11] Burkholder B.M., Jabs D.A. (2021). Uveitis for the non-ophthalmologist. BMJ.

[12] Kalogeropoulos D., Sakkas H., Mohammed B., Vartholomatos G., Malamos K., Sreekantam S., Kanavaros P., Kalogeropoulos C. (2022). Ocular toxoplasmosis: A review of the current diagnostic and therapeutic approaches. Int. Ophthalmol..

[13] Rong S., Yu X., Wiggs J.L. (2024). Genetic Basis of Pigment Dispersion Syndrome and Pigmentary Glaucoma: An Update and Functional Insights. Genes.

[14] Li M., Tian M., Wang Y., Ma H., Zhou Y., Jiang X., Liu Y. (2023). Updates on RPE Cell Damage in Diabetic Retinopathy (Review). Mol. Med. Rep..

[15] Jarrett S.G., Boulton M.E. (2012). Consequences of Oxidative Stress in Age-Related Macular Degeneration. Mol. Asp. Med..

[16] Fung T.H., Patel B., Wilmot E.G., Amoaku W.M. (2022). Diabetic Retinopathy for the Non-Ophthalmologist. Clin. Med..

[17] Tonade D., Kern T.S. (2021). Photoreceptor Cells and RPE Contribute to the Development of Diabetic Retinopathy. Prog. Retin. Eye Res..

[18] Kaarniranta K., Blasiak J., Liton P., Boulton M., Klionsky D.J., Sinha D. (2023). Autophagy in Age-Related Macular Degeneration. Autophagy.

[19] He F., Ru X., Wen T. (2020). NRF2, a Transcription Factor for Stress Response and Beyond. Int. J. Mol. Sci..

[20] Yang Y.-C., Chien Y., Yarmishyn A.A., Lim L.-Y., Tsai H.-Y., Kuo W.-C., Tsai P.-H., Yang S.-H., Hong S.-I., Chen S.-J. (2024). Inhibition of Oxidative Stress-Induced Epithelial-Mesenchymal Transition in Retinal Pigment Epithelial Cells of Age-Related Macular Degeneration Model by Suppressing ERK Activation. J. Adv. Res..

[21] Tan T.-E., Wong T.Y. (2022). Diabetic Retinopathy: Looking Forward to 2030. Front. Endocrinol..

[22] Tatsumi T. (2023). Current Treatments for Diabetic Macular Edema. Int. J. Mol. Sci..

[23] Zhang C., Gu L., Xie H., Liu Y., Huang P., Zhang J., Luo D., Zhang J. (2024). Glucose Transport, Transporters and Metabolism in Diabetic Retinopathy. Biochim. Biophys. Acta Mol. Basis Dis..

[24] Datta S., Cano M., Ebrahimi K., Wang L., Handa J.T. (2017). The Impact of Oxidative Stress and Inflammation on RPE Degeneration in Non-Neovascular AMD. Prog. Retin. Eye Res..

[25] Cornebise C., Perus M., Hermetet F., Vallas-Fonavet J., Richard T., Aires V., Delmas D. (2023). Red Wine Extract Prevents Oxidative Stress and Inflammation in ARPe-19 Retinal Cells. Cells.

[26] Harju N. (2022). Regulation of oxidative stress and inflammatory responses in human retinal pigment epithelial cells. Acta Ophthalmol..

[27] Kushwah N., Bora K., Maurya M., Pavlovich M.C., Chen J. (2023). Oxidative Stress and Antioxidants in Age-Related Macular Degeneration. Antioxidants.

[28] Bungau S., Abdel-Daim M.M., Tit D.M., Ghanem E., Sato S., Maruyama-Inoue M., Yamane S., Kadonosono K. (2019). Health Benefits of Polyphenols and Carotenoids in Age-Related Eye Diseases. Oxid. Med. Cell. Longev..

[29] Lykkesfeldt J. (2007). Malondialdehyde as Biomarker of Oxidative Damage to Lipids Caused by Smoking. Clin. Chim. Acta.

[30] Cano M., Wang L., Wan J., Barnett B.P., Ebrahimi K., Qian J., Handa J.T. (2014). Oxidative Stress Induces Mitochondrial Dysfunction and a Protective Unfolded Protein Response in RPE Cells. Free Radic. Biol. Med..

[31] Lin Y.-H., Sheu S.-J., Liu W., Hsu Y.-T., He C.-X., Wu C.-Y., Chen K.-J., Lee P.-Y., Chiu C.-C., Cheng K.-C. (2023). Retinal Protective Effect of Curcumin Metabolite Hexahydrocurcumin against Blue Light-Induced RPE Damage. Phytomedicine.

[32] Grimm C., Remé C.E., Rol P.O., Williams T.P. (2000). Blue Light’s Effects on Rhodopsin: Photoreversal of Bleaching in Living Rat Eyes. Investig. Ophthalmol. Vis. Sci..

[33] Grimm C., Remé C.E., Weber B.H.F., Langmann T. (2019). Light Damage Models of Retinal Degeneration. Retinal Degeneration.

[34] Kuse Y., Ogawa K., Tsuruma K., Shimazawa M., Hara H. (2014). Damage of Photoreceptor-Derived Cells in Culture Induced by Light Emitting Diode-Derived Blue Light. Sci. Rep..

[35] Ouyang X., Yang J., Hong Z., Wu Y., Xie Y., Wang G. (2020). Mechanisms of Blue Light-Induced Eye Hazard and Protective Measures: A Review. Biomed. Pharmacother..

[36] Talens-Estarelles C., García-Marqués J.V., Cervino A., García-Lázaro S. (2021). Use of Digital Displays and Ocular Surface Alterations: A Review. Ocul. Surf..

[37] Guymer R.H., Campbell T.G. (2023). Age-related mascular degeneration. Lancet.

[38] Llorián-Salvador M., de la Fuente A.G., McMurran C.E., Dashwood A., Dooley J., Liston A., Penalva R., Dombrowski Y., Stitt A.W., Fitzgerald D.C. (2024). Regulatory T Cells Limit Age-Associated Retinal Inflammation and Neurodegeneration. Mol. Neurodegener..

[39] Choudhary M., Malek G. (2023). CD68: Potential Contributor to Inflammation and RPE Cell Dystrophy. Adv. Exp. Med. Biol..

[40] Huang Y., Cao S., Zhang Q., Zhang H., Fan Y., Qiu F., Kang N. (2018). Biological and Pharmacological Effects of Hexahydrocurcumin, a Metabolite of Curcumin. Arch. Biochem. Biophys..

[41] Bharti K., den Hollander A.I., Lakkaraju A., Sinha D., Williams D.S., Finnemann S.C., Bowes-Rickman C., Malek G., D’Amore P.A. (2022). Cell Culture Models to Study Retinal Pigment Epithelium-Related Pathogenesis in Age-Related Macular Degeneration. Exp. Eye Res..

[42] Kuźmińska J., Sobczak A., Wierzchowski M., Gośliński T., Jelińska A. (2021). Efekty działania fitoestrogenów w nowotworach hormonozależnych. Farmacja.

[43] Pandey A., Chaturvedi M., Mishra S., Kumar P., Somvanshi P., Chaturvedi R. (2020). Reductive Metabolites of Curcumin and Their Therapeutic Effects. Heliyon.

[44] Zia A., Farkhondeh T., Pourbagher-Shahri A.M., Samarghandian S. (2021). The Role of Curcumin in Aging and Senescence: Molecular Mechanisms. Biomed. Pharmacother..

[45] Sadeghi M., Dehnavi S., Asadirad A., Xu S., Majeed M., Jamialahmadi T., Johnston T.P., Sahebkar A. (2023). Curcumin and Chemokines: Mechanism of Action and Therapeutic Potential in Inflammatory Diseases. Inflammopharmacology.

[46] Rohilla M., Rishabh Bansal S., Garg A., Dhiman S., Dhankhar S., Saini M., Chauhan S., Alsubaie N., Batiha G.E., Albezrah N.K.A. (2023). Discussing pathologic mechanisms of Diabetic retinopathy & therapeutic potentials of curcumin and β-glucogallin in the management of Diabetic retinopathy. Biomed. Pharmacother..

[47] Yang J., Miao X., Yang F.J., Cao J.F., Liu X., Fu J.L., Su G.F. (2021). Therapeutic potential of curcumin in diabetic retinopathy. Int. J. Mol. Med..

[48] Balasubramanyam M., Koteswari A.A., Kumar R.S., Monickaraj S.F., Maheswari J.U., Mohan V. (2003). Curcumin-induced inhibition of cellular reactive oxygen species generation: Novel therapeutic implications. J. Biosci..

[49] Joshi P., Joshi S., Semwal D.K., Verma K., Dwivedi J., Sharma S. (2022). Role of curcumin in ameliorating hypertension and associated conditions: A mechanistic insight. Mol. Cell. Biochem..

[50] Bianchetti G., Clementi M.E., Sampaolese B., Serantoni C., Abeltino A., De Spirito M., Sasson S., Maulucci G. (2023). Metabolic Imaging and Molecular Biology Reveal the Interplay between Lipid Metabolism and DHA-Induced Modulation of Redox Homeostasis in RPE Cells. Antioxidants.

[51] Chaiyasaeng W., Hongwiset D., Tocharus C., Punyawudho B., Tocharus J., Chaichompoo W., Rojsitthisak P., Pabuprapap W., Yingyongnarongkul B.E., Suksamrarn A. (2024). Comparative Pharmacokinetics and Tissue Distribution of Hexahydrocurcumin Following Intraperitoneal vs Oral Administration in Mice Using LC-MS/MS. ACS Omega.

[52] Kuo C.-N., Chen C.-H., Chen S.-N., Huang J.-C., Lai L.-J., Lai C.-H., Hung C.-H., Lee C.-H., Chen C.-Y. (2018). Anti-Angiogenic Effect of Hexahydrocurcumin in Rat Corneal Neovascularization. Int. Ophthalmol..

[53] Panthiya L., Tocharus J., Onsa-Ard A., Chaichompoo W., Suksamrarn A., Tocharus C. (2022). Hexahydrocurcumin Ameliorates Hypertensive and Vascular Remodeling in L-NAME-Induced Rats. Biochim. Biophys. Acta Mol. Basis Dis..

[54] Jearjaroen P., Thangwong P., Tocharus C., Chaichompoo W., Suksamrarn A., Tocharus J. (2024). Hexahydrocurcumin Attenuated Demyelination and Improved Cognitive Impairment in Chronic Cerebral Hypoperfusion Rats. Inflammopharmacology.

[55] Sudarshan K., Yarlagadda S., Sengupta S. (2024). Recent Advances in the Synthesis of Diarylheptanoids. Chem. Asian J..

[56] Sudarshan K., Perumal G., Aidhen I.S., Doble M. (2018). Synthesis of Unsymmetrical Linear Diarylheptanoids and their Enantiomers and Antiproliferative Activity Studies. Eur. J. Org. Chem..

